# Arzneimitteltherapiesicherheit gefördert durch die interprofessionelle Zusammenarbeit von Arzt und Apotheker auf Intensivstationen in Deutschland: Erkenntnisse einer Umfrage

**DOI:** 10.1007/s00063-022-00898-5

**Published:** 2022-03-08

**Authors:** Heike Hilgarth, Christian Waydhas, Frank Dörje, Julia Sommer, Stefan Kluge, Karl Peter Ittner

**Affiliations:** 1 Klinikapotheke und Klinik für Intensivmedizin, Universitätsklinikum Hamburg-Eppendorf, Hamburg, Deutschland; 2 Ausschuss für Intensivmedizin und klinische Ernährung, ADKA – Bundesverband Deutscher Krankenhausapotheker e. V., Berlin, Deutschland; 3 Sektionsgruppe Qualitätsverbesserung und Informationstechnologie, Deutsche Interdisziplinäre Vereinigung für Intensiv- und Notfallmedizin (DIVI), Berlin, Deutschland; 4 Klinik und Poliklinik für Chirurgie, Berufsgenossenschaftliches Universitätsklinikum Bergmannsheil, Bochum, Deutschland; 5 Medizinische Fakultät, Universität Duisburg-Essen, Essen, Deutschland; 6 Apotheke des Universitätsklinikums Erlangen, Universitätsklinikum Erlangen, Erlangen, Deutschland; 7 Klinik für Intensivmedizin, Universitätsklinikum Hamburg-Eppendorf, Hamburg, Deutschland; 8 Deutsche Interdisziplinäre Vereinigung für Intensiv- und Notfallmedizin (DIVI), Berlin, Deutschland; 9 Lehr- und Forschungseinheit Pharmakologie, Fakultät für Medizin, Universität Regensburg, Regensburg, Deutschland; 10 Klinik für Anästhesiologie, Universitätsklinikum Regensburg, Regensburg, Deutschland

**Keywords:** Pharmazeutische Betreuung, AMTS, Stationsapotheker, Intensivmedizin, Patientensicherheit, Pharmaceutical care, Drug therapy safety, Clinical pharmacist, Critical care, Patient safety

## Abstract

**Hintergrund:**

Kritisch kranke Patienten sind besonders anfällig für unerwünschte Arzneimittelereignisse. Internationale Studien zeigen, dass pharmazeutische Betreuung die Patienten- und Arzneimitteltherapiesicherheit positiv beeinflusst. National wird die Integration von Apothekern in das multidisziplinäre Team und eine Teilnahme an Visiten gefordert. Ziel dieser Arbeit ist es, Art und Umfang der pharmazeutischen Betreuung in der Intensivmedizin in Deutschland zu erheben.

**Methode:**

In einer Literatur- und Datenbankrecherche wurden 13 relevante pharmazeutische Tätigkeiten identifiziert. Darauf aufbauend wurde von einem Expertengremium ein Onlinesurvey mit 27 Fragen zur Implementierung der pharmazeutischen Betreuung auf Intensivstationen erstellt. Die Umfrage wurde an Leiter deutscher Intensivstationen versandt.

**Ergebnisse:**

Eine regelmäßige pharmazeutische Betreuung ist bei 35,3 % (59/167) der Intensivstationen etabliert. Arzneimittelinformation (89,7 % [52/58]), pharmazeutische Interventionen mit Therapieumstellung (z. B. in der Visite; 67,2 % [39/58]), regelmäßige Evaluation der Verordnung (Medikationsanalyse; 65,5 % [38/58]) sowie die Überwachung der Medikation (hinsichtlich von Nebenwirkungen, Effektivität und Kosten; 63,8 % [37/58]) zählen zu den meistgenannten Tätigkeiten. Die Teilnehmer mit pharmazeutischer Betreuung (58/168) stufen 7 von 13 Tätigkeiten als „essenziell/unverzichtbar“ ein, wohingegen es nur zwei bei den Teilnehmern ohne pharmazeutische Betreuung (104/168) sind.

**Schlussfolgerung:**

Nur wenige Intensivstationen in Deutschland haben den Stationsapotheker bereits in das multidisziplinäre Team integriert. Ist ein pharmazeutischer Service etabliert, wird mehreren pharmazeutischen Tätigkeitsfeldern eine höhere Gewichtung/Bedeutung zugeschrieben.

**Zusatzmaterial online:**

Zusätzliche Informationen sind in der Onlineversion dieses Artikels (10.1007/s00063-022-00898-5) enthalten.

## Abstract (en)

**Background:**

Critically ill patients are particularly susceptible to adverse drug events. International studies show that pharmaceutical care has a positive impact on patient and drug therapy safety. Nationally, the integration of pharmacists into the multidisciplinary team and participation in ward rounds is required. The aim of this work is to assess the scope and extent of pharmaceutical care in intensive care units (ICU) in Germany.

**Method:**

In a literature and database search, 13 relevant pharmaceutical activities were identified. Based on this, an online survey with 27 questions on the implementation of pharmaceutical care in ICU was prepared by a panel of experts. The survey was sent to heads of German ICUs.

**Results:**

Of the participants, 35.3% (59/167) have established regular pharmaceutical care. Drug information (89.7% [52/58]), pharmaceutical interventions with change of therapy (e.g., ward rounds; 67,2% [39/58]), regular evaluation of prescriptions (medication analysis; 65.5% [38/58]) as well as the monitoring of medication (e.g., side effects, effectiveness, costs; 63.8% [37/58]) were most frequently mentioned. The participants with pharmaceutical care (58/168) graded 7 of 13 but those without (104/168) only two  activities as ‘essential/indispensable’.

**Conclusion:**

Only a few ICU in Germany have already integrated ward pharmacists into the multidisciplinary team. Once a pharmaceutical service has been established, a greater role/importance is assigned to several pharmaceutical activities.

## Einleitung

Die Arzneimitteltherapie von kritisch kranken Patienten ist geprägt durch Polypharmazie, Organinsuffizienzen und den Einsatz modernster Organersatzverfahren. Diese hochkomplexen Therapieregime erhöhen das Risiko für unerwünschte Arzneimittelereignisse (UAE) und Medikationsfehler (MF). Durchschnittlich treten bei 10 kritisch kranken Patienten jeden Tag 0,8 UAE und 1,5 MF auf, sodass klinische Studien eine Inzidenz von 106 MF an 1000 Patiententagen (Median) berichten [14, 23]. UAE und MF werden zusammen mit Nebenwirkungen unter dem Begriff arzneimittelbezogene Probleme (ABP) subsumiert. ABP können das Erreichen angestrebter Therapieziele verhindern und an jeder Stelle des Medikationsprozesses auftreten [2, 28]. In der Folge kann es neben einer Verlängerung der Liegedauer auch häufiger zu einem dauerhaften Patientenschaden, dem Einsatz von lebenserhaltenen Maßnahmen und/oder Tod (3,7 %) als auf Nicht-Intensivstationen (1,9 %) führen [14]. Eine Integration von Apothekern ins Team kann die Patienten- und Arzneimitteltherapiesicherheit verbessern und wird sowohl international als auch national empfohlen und z. T. auch gesetzlich forciert [9, 13, 15, 26]. Die Deutsche Interdisziplinäre Vereinigung für Intensiv- und Notfallmedizin (DIVI) fordert seit 2010 die Teilnahme von Apothekern an den Visiten auf Intensivstation mindestens einmal wöchentlich sowie eine kontinuierliche konsiliarische Verfügbarkeit [11]. Apotheker analysieren die verordnete Medikation patientenindividuell und können zur Optimierung der Therapie und zur Arzneimitteltherapiesicherheit (AMTS) beitragen [25]. Dies beeinflusst klinisch relevante Parameter (Mortalität und Liegedauer) positiv und reduziert Kosten [4, 17, 18, 22, 30]. Zur Prävention von ABP sind in der Literatur verschiedene Maßnahmen als geeignet beschrieben worden. Dazu zählen: Implementierung einer regelmäßigen pharmazeutischen Betreuung (z. B. Medikationsanalysen, Visiten), Etablierung von Standards, regelmäßige Schulungen des medizinischen Personals und die Einführung von Computerized-physician-order-entry(CPOE)-Systemen [8].

Im englischsprachigen Raum sind Apotheker bereits fester Bestandteil des multiprofessionellen Teams und übernehmen Verantwortung entsprechend ihrer Funktion und Qualifikation [10, 13, 17, 19, 26]. Inwiefern Apotheker auf deutschen Intensivstationen tätig sind, ist derzeit nicht bekannt. *Ziel* dieser Untersuchung ist es, die Art und den Umfang der pharmazeutischen Betreuung auf deutschen Intensivstationen zu erheben. Darüber hinaus werden verschiedene pharmazeutische Tätigkeitsfelder evaluiert und hinsichtlich ihrer Bedeutung kategorisiert.

## Methode

### Literaturrecherche

Nach den Kriterien „finding current best evidence“ aus JAMAevidence [1] wurden internationale Evidence-best-medicine(EBM)-Datenbanken und „systematic reviews“ nach „pharmacy“ AND „critical care“ durchsucht. Diese Kriterien wurden auch einer Schlagwortsuche in der medizinischen Onlinebibliothek (McGrawHill) und PubMed zugrundegelegt.

### Elektronische Umfrage

Ein Expertengremium (bestehend aus Apothekern und Intensivmedizinern) entwickelte basierend auf den Literaturrecherche einen Fragebogen mit 27 Fragen (s. Online-Zusatzmaterial).

Neben der Erhebung allgemeiner Daten (10 Fragen) wurden vor allem Fragen zu Art und Umfang der pharmazeutischen Betreuung (16 Fragen) gestellt. Abschließend wurde eine Wichtung („essenziell/unverzichtbar“, „wünschenswert“ oder „optional“) von 13 pharmazeutischen Tätigkeiten von allen Teilnehmern durchgeführt. Für die Beantwortung der Fragen waren Mehrfachantworten, Freitexteingaben sowie Nichtbeantwortung von Fragen zulässig.

Im Juli 2019 wurden E‑Mails mit dem Link zum Fragenbogen (survey-monkey®, https://www.surveymonkey.com; San Mateo, CA, United States) an 1549 Leiter deutscher Intensivstationen über die DIVI datenschutzkonform versendet. Ein Reminder wurde im September 2019 versandt. Alle Antworten, die bis zum 31.10.2019 eingingen, wurden berücksichtigt. Die Analyse der pseudonymisierten Daten erfolgte deskriptiv mithilfe von Microsoft Excel (Microsoft Office 365, Version 1810, Redmond, WA, USA).

Die Untersuchung wurde der Ethikkommission der Ärztekammer Hamburg vorgelegt, eine Zustimmung war aber nicht erforderlich.

## Ergebnisse

### Ergebnisse der Literaturrecherche

Alle durchsuchten Literaturquellen ergaben, dass die klinische Pharmazie und der Stationsapotheker auf einer Intensivstation international fest etabliert sind [6, 10, 13, 16, 18, 20, 27] (s. Online Zusatzmatrial).

### Demographie

Die Rücklaufquote betrug 11 % (168/1549). Es haben vor allem Vertreter der Regel- (44 % [74/168]) und Schwerpunktversorgung (32 % [54/168]) an der Umfrage teilgenommen mit einem geringeren Rücklauf aus der Maximalversorgung (24 % [40/168]). Die Umfrage wurde mehrheitlich von Chefärzten (56 % [94/168]) oder Oberärzten (42 % [71/168]) und nur selten durch andere Berufsgruppen (2 % [3/168]) beantwortet. Demographische Angaben der teilnehmenden Krankenhäuser sind in Tab. 1 dargestellt.

**Table 1:** 

	Alle Teilnehmer (*n* = 168)	Teilnehmer (*n* = 59) mit pharmazeutischer Betreuung
**Bettenanzahl**		
<400 Betten	76 (45,2 %)	27 (45,8 %)
400–800 Betten	54 (32,1 %)	18 (30,5 %)
>800 Betten	38 (22,6 %)	14 (23,7 %)
**Versorgungsstufe**		
Regelversorgung	74 (44,1 %)	25 (42,3 %)
Schwerpunktversorgung	54 (32,1 %)	20 (33,9 %)
Maximalversorgung	40 (23,8 %)	14 (23,7 %)
**Anzahl der Intensiv‑/Intermediate Care Stationen im Krankenhaus der Teilnehmer**		
1	67 (39,9 %)	24 (40,7 %)
2	43 (25,6 %)	12 (20,3 %)
3	15 (8,9 %)	5 (8,5 %)
Mehr als 3	43 (25,6 %)	18 (30,5 %)
**Qualifikation der Teilnehmer**		
Chefarzt	94 (56,0 %)	35 (59,3 %)
Oberarzt	71 (42,3 %)	23 (39,0 %)
Facharzt	–	–
Assistenzarzt	–	–
Andere	3 (1,8 %)	1 (1,7 %)
**Arbeitsbereich (Anzahl der Teilnehmer)**		
Bereich Erwachsene (gesamt)	217	73
*Intensivstation*	150	53
*Intermediate-Care-Station*	67	20
Bereich Kinder (gesamt)	23	7
*Intensivstation*	17	5
*Intermediate-Care-Station*	6	2

### Ergebnisse zum aktuellen Stand der pharmazeutischen Betreuung

**Figure 1:**
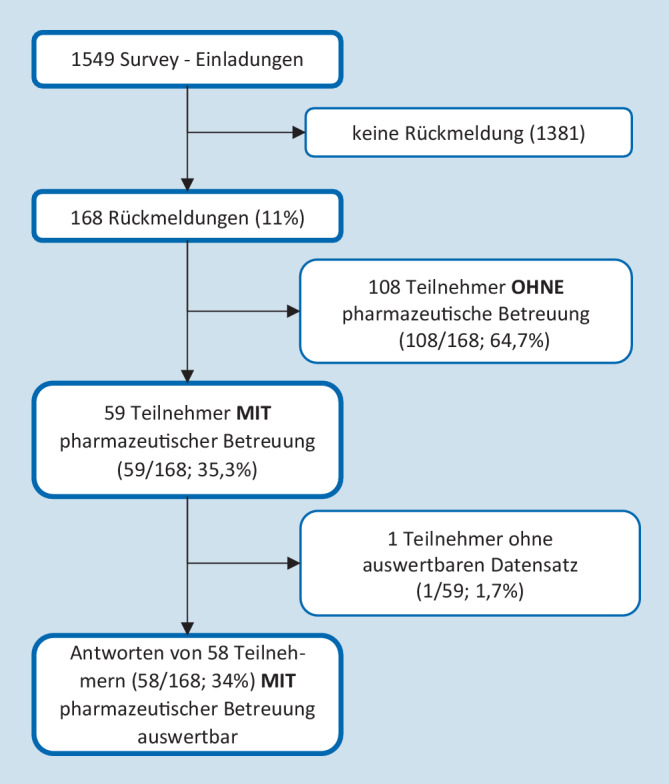

Eine *regelmäßige pharmazeutische Betreuung ist bei 35,3* *%* (59/167) der Intensivstationen etabliert (s. Abb. 1). Dieser Anteil variiert innerhalb der verschiedenen Versorgungsstufen (Regelversorgung 42,3 % [25/59], Schwerpunktversorgung 33,9 % [20/59], Maximalversorgung 23,7 % [14/59]; s. Tab. 1).

### Art der pharmazeutischen Tätigkeiten auf der Intensivstation

**Figure 2:**
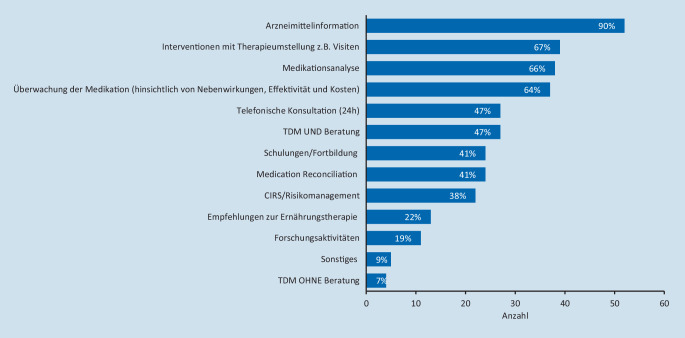

In der Gruppe der* Teilnehmer mit pharmazeutischer Betreuung *ist die Arzneimittelinformation durch Apotheker bei fast allen implementiert (89,7 % [52/58]). Häufig wurden pharmazeutische Interventionen mit Therapieumstellung (z. B. in der Visite; 67,2 % [39/58]) und einer regelmäßigen Evaluation der Verordnung (Medikationsanalyse; 65,5 % [38/58]) sowie die Überwachung der Medikation (hinsichtlich von Nebenwirkungen, Effektivität und Kosten; 63,8 % [37/58]) genannt. Darüber hinaus sind die telefonische Konsultation über 24 h (46,6 % [27/58]), therapeutisches Drugmonitoring (TDM) inkl. Beratung (46,6 % [27/58]), Schulungen/Fortbildungen durch und mit Apothekern (41,4 % [24/58]) sowie die Überprüfung der Medikation auf Vollständigkeit („medication reconciliation“; 41,4 % [24/58]) etablierte Tätigkeiten. Die Einbindung in das CIRS/Risikomanagement findet bei 37,9 % (22/58) statt. Weniger häufig werden Empfehlungen zur Ernährungstherapie (22,4 % [13/58]), Forschungsaktivitäten (19,0 % [11/58]), TDM ohne Beratung (6,9 % [4/58]) sowie sonstiges (z. B. Antibiotic Stewardship; 8,6 % [5/58]) angegeben (s. Abb. 2).

### Umfang der pharmazeutischen Tätigkeiten auf der Intensivstation

*Visiten mit Apothekern* finden überwiegend „wöchentlich“ (62,1 % [36/58]) und seltener „auf Anfrage“ (17,2 % [10/58]), „2- bis 3‑mal wöchentlich“ oder „täglich“ (je 5,2 % [3/58]) bzw. gar nicht (12,1 % [7/58]) statt. *Ansprechpartner für Apotheker* sind primär Oberärzte (91,4 % [53/58]), gefolgt von Fach- (41,4 % [24/58]) und Assistenzärzten (39,7 % [23/58]) sowie Pflegefachkräften (32,8 % [19/58]) und Chefärzten (27,6 % [16/58]). Die Kommunikation erfolgt vor allem telefonisch (67,2 % [39/58]) oder „mündlich ohne schriftliche Dokumentation“ (z. B. in Visiten; 63,8 % [37/58]) und zu einem geringeren Anteil „mündlich und schriftlich in der Patientenakte“ (32,8 % [19/58]) bzw. schriftlich (z. B. Konsile; 15,5 % [9/58]). Die *Berufserfahrung *der Apotheker wird mehrheitlich auf mindestens 10 Jahre (43,1 % [25/58]) und seltener auf 5–10 Jahre (29,3 % [17/58]) bzw. weniger als 5 Jahre (5,2 % [3/58]) geschätzt. Selten sind Apotheker nur der Intensivstation (8,6 % [5/58]) zugeordnet. Regelhaft übernehmen sie auch andere Aufgaben und Funktionen (89,7 % [52/58]).

### Wichtung der pharmazeutischen Tätigkeiten auf der Intensivstation

Teilnehmer (58/168) *mit etablierter pharmazeutischer Betreuung* gewichten 7 von 13 Tätigkeitsfeldern als „essenziell/unverzichtbar“. Dies schließt die Bereitstellung von Arzneimittelinformation (74,1 % [43/58]), Überwachung der Medikation (hinsichtlich Nebenwirkungen, Effektivität und Kosten; 51,7 % [30/58]), TDM und Beratung (50,0 % [28/56]), regelmäßige Evaluation der Verordnung (Medikationsanalyse; 50,0 % [29/58]) sowie die Teilnahme an Visiten (50,0 % [29/58]) und telefonische Erreichbarkeit (42,9 % [24/56]) ein. Die Einbindung in das CIRS/Risikomanagement (je 42,1 % [24/57]) wird zu gleichen Teilen als „essenziell/unverzichtbar“ und „wünschenswert“ bewertet. Als „wünschenswert“ sind die Teilnahme und Durchführung von Schulungen/Fortbildungen (61,4 % [35/57]), Überprüfung der Medikation auf Vollständigkeit („medication reconciliation“; 52,6 % [30/57]), Empfehlungen zur Ernährungstherapie (46,4 % [26/56]) und Interventionen, die zur Therapieumstellung (Eskalation, Deeskalation) führen (42,9 % [24/56]) kategorisiert.

Teilnehmer *ohne pharmazeutische Betreuung* (104/168) bewerten nur die Bereitstellung von Arzneimittelinformation (52,9 % [54/102]) und die telefonische Konsultation über 24 h (42,3 % [44/104]) als „essenziell/unverzichtbar“. Neun weitere werden als „wünschenswert“ eingeschätzt und beinhalten die Teilnahme an Visiten (63,5 % [66/104]), Überwachung der Medikation hinsichtlich Nebenwirkungen, Effektivität und Kosten (63,5 % [66/104]), Teilnahme und Durchführung von Schulungen/Fortbildungen (61,5 % [64/104]), Überprüfung der Medikation auf Vollständigkeit („medication reconciliation“; 60,2 % [62/103]), Interventionen, die zur Therapieumstellung (Eskalation, Deeskalation) führen (59,6 % [62/104]), Einbindung in das CIRS/Risikomanagement (59,6 % [62/104]), TDM und Beratung (55,3 % [57/103]), regelmäßige Evaluation der Verordnung (Medikationsanalyse; 50,0 % [52/104]) sowie Empfehlungen zur Ernährungstherapie (49,0 % [51/104]).

Übereinstimmend wichten *alle Teilnehmer* Forschungsaktivitäten (54,9 % [28/51] bzw. 62,8 % [64/102]) und TDM ohne Beratung (56,5 % [26/46] bzw. 47,8 % [44/92]) als „optional“ (s. Tab. 2).

**Table 2:** 

Pharmazeutische Tätigkeiten	Alle Teilnehmer /Teilnehmer ohne pharmazeutsiche Betreuung	Teilnehmer mit pharmazeutischer Betreuung
Bereitstellung von Arzneimittelinformation	Essenziell/unverzichtbar	Essenziell/unverzichtbar
Telefonische Erreichbarkeit (24 h)	Essenziell/unverzichtbar	Essenziell/unverzichtbar
Teilnahme an Visiten	Wünschenswert	Essenziell/unverzichtbar
Regelmäßige Evaluation der Verordnung (Medikationsanalyse)	Wünschenswert	Essenziell/unverzichtbar
Überwachung der Medikation (hinsichtlich Nebenwirkung, Effektivität, Kosten)	Wünschenswert	Essenziell/unverzichtbar
TDM und Beratung	Wünschenswert	Essenziell/unverzichtbar
Einbindung in das CIRS/Risikomanagement	Wünschenswert	Essenziell/unverzichtbar bzw. wünschenswert
Interventionen, die zur Therapieumstellung führen	Wünschenswert	Wünschenswert
Überprüfung der Medikation auf Vollständigkeit („medication reconciliation“)	Wünschenswert	Wünschenswert
Empfehlungen zur Ernährungstherapie	Wünschenswert	Wünschenswert
Die Teilnahme und Durchführung von Schulungen/Fortbildungen	Wünschenswert	Wünschenswert
Forschungsaktivitäten	Optional	Optional
TDM ohne Beratung	Optional	Optional

### Elektronische Akte

36,9 % (62/168) aller Befragten arbeiten papierlos mit einem *elektronischen Verordnungssystem*. Aufgrund der Möglichkeit zur Mehrfachauswahl ergibt sich ein Anteil von 38,7 % (24/62), die eine elektronische Verordnung auf allen Stationen nutzen, demgegenüber stehen 50 % (31/62) „nur auf Intensivstationen“ bzw. 17,7 % (11/62) „auf einzelnen Stationen“.

## Diskussion

Erstmals liegen für Deutschland Ergebnisse zu Art und Umfang der pharmazeutischen Betreuung auf der Intensivstation vor. 35,3 % der Teilnehmer haben eine regelmäßige pharmazeutische Betreuung etabliert, wohingegen dies im internationalen Vergleich 70,8–98,6 % der Intensivstationen sind [6, 19].

Die PROTECTED-UK-Studie zeigte, dass pharmazeutische Interventionen in 73,8 % zur Optimierung der Wirksamkeit und/oder zur AMTS beitragen und vor allem in Visiten (59,4 %) diskutiert werden [18]. In einer kanadischen Umfrage waren die Evaluation der Medikation und Dosierung die häufigsten Interventionen [20]. Auch in der vorliegenden Untersuchung sind Arzneimittelinformation, die Teilnahme an Visiten, die regelmäßige Evaluation und Überwachung der Medikation sowie das TDM inkl. Beratung durch Apotheker wichtige Bestandteile pharmazeutischer Betreuung. Für Deutschland wurde kürzlich gezeigt, dass tägliche Visitenteilnahmen und die Durchführung von Medikationsanalysen sowie regelmäßige Schulungen signifikant MF (u. a. falsche Dosis, fehlende Dosisanpassung, Übertragungsfehler etc.) reduzieren (14,12 % auf 3,25 %) [12]. In britischen Empfehlungen werden die Medikationsanalyse, eine mindestens werktägliche Teilnahme an Visiten, die Mitarbeit bei der Erstellung von Therapieempfehlungen sowie im Risiko- und Qualitätsmanagement zu den Kernaufgaben von Apothekern auf Intensivstation gezählt [26]. Eine intensivmedizinische Visite mit Apothekern findet in Deutschland noch vorwiegend wöchentlich (62,1 %) statt. Dies ist konform zur aktuellen Empfehlung der DIVI [11] und wird auch von einer deutschen Untersuchung aus dem Jahr 2017 bestätigt [24]. Nur 5,2 % (3/58) berichten über eine tägliche Visite, die in den USA und Großbritannien als „essenzielle“ Tätigkeit fest etabliert ist [6, 19]. In den aktuellen amerikanischen Empfehlungen werden 82 pharmazeutische Tätigkeiten auf der Intensivstation charakterisiert und in „essenziell“ und „wünschenswert“ kategorisiert [13]. Die Rolle des Apothekers wird in 5 verschiedenen Bereichen (direkte Patientenbetreuung, Qualitätssteigerung, Forschung/Wissenschaft, Ausbildung/Lehre sowie kontinuierliche berufliche Weiterentwicklung) detailliert beschrieben [13].

Neben der Ermittlung des Ist-Zustands wurde der Stellenwert der pharmazeutischen Tätigkeiten für das medizinische Personal untersucht. Dies ist besonders relevant, da sich daraus wichtige Kooperationsbereiche bzw. Tätigkeitsfelder für Apotheker ableiten lassen. Insgesamt werden 11 von 13 pharmazeutischen Tätigkeitsfeldern entweder als „essenziell/unverzichtbar“ oder als „wünschenswert“ eingestuft. Während in der Gruppe ohne pharmazeutische Betreuung nur zwei Tätigkeiten von allen Teilnehmern als „essenziell/unverzichtbar“ eingestuft wurden, sind es weitere 5 in der Gruppe mit pharmazeutischer Betreuung. Insbesondere die Gruppe, die bereits Erfahrungen mit klinisch-pharmazeutischer Betreuung hat, unterstreicht die Bedeutung des Apothekers im Team.

Direkte und proaktive pharmazeutische Dienstleistungen tragen zur Erhöhung der Patienten- und Arzneimitteltherapiesicherheit sowie zur Reduktion von Liegedauer und Mortalität bei [15, 17, 30]. Diese Dienstleistungen finden in Deutschland nicht nur auf Intensivstationen sondern auch auf Nicht-Intensivstationen statt [24].

Eine elektronische Verordnungssoftware ist nur in 36,9 % (62/168) bei allen bzw. bei 37,3 % (22/59) der Teilnehmer mit pharmazeutischer Betreuung etabliert. Eine deutsche Untersuchung aus 2019 bestätigt diese Ergebnisse (32 %) [22]. Demgegenüber verfügten bereits 2015 84 % der Kliniken in den USA über elektronische Verordnungssysteme (CPOE) [21]. CPOE mit klinischem Entscheidungsunterstützungssystem (CDSS) reduzieren Fehler im gesamten Medikationsprozess [29]. Darüber hinaus identifizieren Stationsapotheker und CDSS unterschiedliche ABP und tragen somit zusätzlich zur Reduktion von ABP bei [31]. Die von der Bundesregierung unterstützte Etablierung eines Closed-loop-medication-management(CLMM)-Prozesses [5, 7] beinhaltet neben elektronischer Verordnung und Dokumentation der Arzneimittelverabreichung interprofessionelles Medikationsmanagement mit Stationsapothekern und eine patientenindividuelle Arzneimittellogistik, z. B. „unit dose“. Diese systematische Unterstützung des Medikationsprozesses als Bestandteil des CLMM verbessert die Patienten- und Arzneimitteltherapiesicherheit [5]. Die Unit-dose-Logistik war aber nur bei 17,3 % (28/162) bzw. 20,7 % (12/58) aller Teilnehmer bzw. derer mit einer pharmazeutischen Betreuung zumindest teilweise etabliert.

Die Ergebnisse der Literaturuntersuchungen definieren die klinische Pharmazie auf einer Intensivstation als elementaren, strukturellen und unverzichtbaren Baustein zur Gewährleistung von Patienten- und Arzneimitteltherapiesicherheit sowie der Qualitätssteigerung [6, 10, 13, 16, 18, 20, 27]. Die im internationalen Vergleich als „essenziell/unverzichtbar“ bzw. „wünschenswert“ bezeichneten pharmazeutischen Tätigkeitsfelder sollten im nationalen Kontext betrachtet werden. Eine Evaluation der unterschiedlichen Strukturen in den Gesundheitssystemen, der universitären Qualifikation sowie den verfügbaren Weiterbildungsmöglichkeiten für Apotheker müssen hierbei berücksichtigt werden. Während in den USA und Großbritannien strukturierte Qualifizierungsprogramme für Apotheker auf der Intensivstation etabliert sind, stehen wir in Deutschland bei Erstellung und Durchführung solcher Maßnahmen noch am Anfang. In Großbritannien verfügen 43,8 % und in den USA 54 % der Apotheker über erweiterte Kenntnisse im Monitoring und in der Bewertung der Pharmakotherapie von Intensivpatienten [6, 19]. Die PROTECTED-UK-Studie zeigte, dass erfahrene und speziell geschulte Apotheker nicht nur häufiger intervenierten, sondern auch mit einer signifikant höheren klinischen Relevanz [25]. Die fachliche Qualifikation und Erfahrung der Apotheker scheinen von entscheidender Bedeutung zu sein. Für Apotheker auf Station gibt es in Deutschland mit dem Fachapotheker für klinische Pharmazie und der Bereichsweiterbildung Medikationsmanagement im Krankenhaus bereits grundlegende Weiterbildungen. Ein zukünftiges Ziel muss es sein, ein kompetenzbasiertes Weiterbildungscurriculum für eine qualitätsgesicherte pharmazeutische Betreuung auch für den Bereich der Intensivmedizin zu entwickeln. Die vorliegende Umfrage liefert wertvolle Hinweise hierzu.

### Limitationen

Der sehr geringe Rücklauf limitiert die Aussagekraft der Erhebung. Als Ursachen können die fehlende Zeit, mangelndes Interesse und häufige Umfragen verantwortlich sein. Eine Verzerrung der Ergebnisse ist insofern nicht auszuschließen, als bei Intensivmedizinern mit etablierter pharmazeutischer Betreuung von einer höheren Bereitschaft/Motivation zur Teilnahme auszugehen ist. Ebenso hätten Apotheker die Ärzte auf die Umfrage hingewiesen haben können. Diese Annahme wird auch von einer aktuellen Studie gestützt [3]. Kliniken ohne regelmäßige pharmazeutische Betreuung fühlten sich ggf. nicht angesprochen, an der Umfrage teilzunehmen. Darüber hinaus kann nicht ausgeschlossen werden, dass die 2019 erhobenen Daten nicht mehr der aktuellen Situation in den Kliniken entsprechen.

## Resümee

Aus dieser Umfrage gehen erstmals Erkenntnisse zu Art und Umfang der pharmazeutischen Betreuung von Intensivstatioenn in Deutschland hervor. Ferner bietet die Umfrage Anlass, strukturierte Weiterbildungen für Apotheker gezielt im intensivmedizinischen Sektor zu evaluieren, zu erarbeiten und zu implementieren. Dies erweitert die pharmazeutische Kompetenz und ist für eine qualitätsgesicherte pharmazeutische Betreuung unerlässlich. Aus der Umfrage geht der klare Wunsch der Intensivmediziner nach einer durchgehenden telefonischen Konsultation sowie deren Wertschätzung der bereitgestellten Arzneimittelinformation hervor. Ärzte, die bereits im Routinebetrieb mit Apothekern zusammenarbeiten, schätzen das gesamte Portfolio an pharmazeutischer Expertise. Noch ist die Umsetzung der DIVI-Empfehlungen aus dem Jahr 2010 nicht flächendeckend, sodass die daraus resultierenden Vorteile in der Patienten- und Arzneimitteltherapiesicherheit noch nicht vollständig realisiert werden. Die Integration von Apothekern in der Intensivmedizin sollte weiter vorangetrieben werden. Die personelle Ausstattung mit Apothekern auf Station wird in der aktuellen Krankenhauserlössituation finanziell nicht gefördert. Ähnlich wie in anderen Bereichen (z. B. Krankenhaushygiene) sollten im Krankenhausentgeltgesetz zukünftig finanzielle Förderregelungen geschaffen werden. Die aktuellen AMTS-Maßnahmen in Deutschland [5] empfehlen die Einführung eines CLMM zur Optimierung des Arzneimitteltherapieprozesses. Dies bedingt weitere Investitionen in pharmazeutisches Personal und in die Digitalisierung der Arzneimittelverordnung und -logistik.

## Fazit für die Praxis

- Nationale und internationale Empfehlungen beschreiben die klinischen Tätigkeitsfelder für Stationsapotheker.
- Pharmazeutische Betreuung ist auf deutschen Intensivstationen noch unzureichend implementiert.
- Intensivstationen mit pharmazeutischer Betreuung bewerten den Apotheker in 7 von 13 abgefragten Tätigkeitsfeldern als unverzichtbar.
- Intensivstationen, bei denen ein Apotheker fest in das multiprofessionelle Team integriert ist, geben den pharmazeutischen Tätigkeiten einen wesentlich höheren Stellenwert in der Patientenversorgung als Intensivstationen ohne pharmazeutischen Service.
- Der Stationsapotheker ist ein wichtiger Partner in der Optimierung und Sicherstellung eines optimalen Medikationsprozesses.

### Supplementary Information





