# Supplementary material for: Drug therapy safety supported by interprofessional collaboration between ICU physicians and clinical pharmacists in critical care units in Germany: Results of a survey
Source: Med Klin Intensivmed Notfmed. 2022 Mar 8;118(2):141–8. [Article in German] doi: 10.1007/s00063-022-00898-5 (PMC9992023; doi:10.1007/s00063-022-00898-5)
Supplement: Supplementary file 1 [file 63_2022_898_MOESM1_ESM.pdf]

Apotheker/innen auf Intensivstation

Willkommen zur Umfrage:
Stationsapotheker/innen auf deutschen Intensivstationen

Sehr geehrte Damen und Herren,

die Sektion Qualitätssicherung der Deutschen Interdisziplinären Vereinigung für Intensiv- und Notfallmedizin (DIVI) und der Ausschuss für Intensivmedizin und klinische Ernährung des Bundesverbandes Deutscher Krankenhausapotheker e.V. (ADKA) führen gemeinsam diese Umfrage zur Tätigkeit der Apotheker/innen auf deutschen Intensivstationen durch und bitten um Ihre Unterstützung.
Pharmazeutische Interventionen (PI) tragen zur Arzneimitteltherapie- und Patientensicherheit im Krankenhaus bei. Der positive Nutzen der Apotheker auf einer Intensivstation bezogen auf die Reduktion von Medikationsfehlern und vermeidbaren unerwünschten Arzneimittelereignissen sowie den damit assoziierten Kosten, als auch der positive Einfluss auf klinische Parameter wie z.B. Mortalität und Aufenthaltsdauer sind in der internationalen Literatur hinreichend veröffentlicht. Daneben sind die Krankenhausapotheker/innen ein wichtiges und geschätztes Mitglied des interdisziplinären Teams einer Intensivstation.
Die in der internationalen Literatur veröffentlichten hohen Akzeptanzraten (74%- 99%) von PI unterstützten diese Aussage.
Bereits im Jahr 2000 erfolgte eine detaillierte Beschreibung der Tätigkeitsfelder der Stationsapotheker in der Intensivmedizin in den USA. Die DIVI Empfehlungen zur Struktur und Ausstattung von Intensivstationen empfehlen die Aufnahme ins interprofessionelle Team und fordern die Teilnahme der Apotheker mindestens einmal wöchentlich an der Visite (Empfehlungsgrad 1B) sowie eine konsiliarische Verfügbarkeit (Empfehlungsgrad 1C). Derzeit liegen für Deutschland keine Daten zum Einsatz von Apotheker/innen auf Intensivstationen und zur Umsetzung dieser Empfehlung der DIVI vor.
Gemeinsam mit der ADKA überprüft die DIVI mit dieser Umfrage die Umsetzung dieser Empfehlung und bestimmt das Ausmaß der Beteiligung der Apotheker im klinischen Alltag auf Intensivstationen. Bitte setzen Sie sich – falls zur Beantwortung der Fragen erforderlich – mit Ihren Apotheker/innen in Verbindung.
Die Ergebnisse der Umfrage werden durch die DIVI und die ADKA ausgewertet und veröffentlicht. Alle Angaben des Fragebogens werden anonymisiert erhoben. Die Auswertung aller Daten erfolgt vollständig anonymisiert ausschließlich durch die beteiligten Berufsverbände. Es erfolgt keine Weitergabe Ihrer E-Mail-Adresse an Dritte. Nach Abschluss der Datenauswertung erhalten Sie eine Analyse der Daten, bezogen auf die Versorgungsstufe des Krankenhauses.

gez.
H.Hilgarth
(Fachapothekerin für Klinische Pharmazie)
i.A. der Sektion Qualitätssicherung der Deutschen Interdisziplinären Vereinigung für Intensiv- und Notfallmedizin (DIVI)
und der Ausschuss für Intensivmedizin und klinische Ernährung des Bundesverbandes Deutscher Krankenhausapotheker e.V. (ADKA)

Apotheker/innen auf Intensivstation

Allgemeine Daten zum Krankenhaus

* 1. Ihr Krankenhaus gehört der folgenden Versorgungsstufe an:

- ☐ Regelversorgung
- ☐ Schwerpunktversorgung
- ☐ Maximalversorgung

* 2. Welcher Berufsgruppe gehören Sie an?

- ☐ Chefärztin/Chefarzt
- ☐ Fachärztin/Facharzt
- ☐ andere
- ☐ Oberärztin/Oberarzt
- ☐ Assistenzärztin/Assistenzarzt

* 3. Wie viele Betten hat Ihr Krankenhaus?

- ☐ < 400
- ☐ 400 - 800
- ☐ > 800

4. Für wie viele Intensiv-Betten sind Sie verantwortlich?

____

5. Für wie viele IMC-Betten sind Sie verantwortlich?

____

* 6. Wie viele Intensiv-/ IMC-Stationen gibt es in Ihrem Haus?

- ☐ eine
- ☐ zwei
- ☐ drei
- ☐ mehr als 3

* 7. In welchem Bereich sind Sie tätig?

- ☐ Intensivmedizin (Erwachsene)
- ☐ Intensivmedizin (Kinder)
- ☐ Intermediate Care Station (Erwachsene)
- ☐ Intermediate Care Station (Kinder)

* 8. Haben Sie ein elektronisches Verordnungssystem, in dem Sie täglich papierlos verordnen?

- ☐ JA
- ☐ NEIN

Apotheker/innen auf Intensivstation

Elektronische Akte

Diese Fragen werden nur von Krankenhäusern beantwortet, die über ein elektronisches Verordnungssystem verfügen.

9. Ein elektronisches Verordnungssystem ist in Ihrem Krankenhaus auf ...

- [ ] allen Stationen aktiv
- [ ] nur im Bereich der Intensivmedizin verfügbar
- [ ] auf einzelnen Stationen aktiv

10. Verfügt Ihr elektronisches Verordnungssystem über eine oder mehrere der unten aufgeführten Funktionen?

	JA	NEIN	Weiß ich nicht
Standardisierte Allergiewarnung	○	○	○
automatischen Interaktionscheck der verordneten Medikamente	○	○	○
automatische Warnung bei Überschreitung der Maximaldosis	○	○	○
automatische Warnung bei Doppelverordnung	○	○	○
automatische Hinweise zur Dosisanpassung (Nieren- und Leberfunktionsstörung)	○	○	○
automatische Warnung bei Inkompatibilität parenteraler Zubereitungen	○	○	○

Apotheker/innen auf Intensivstation

Apotheker/innen auf der Intensivstation

Diese Seite bezieht sich auf die Tätigkeit der Apotheker/innen auf der Intensivstation bzw. umfasst Fragen zur pharmazeutischen Betreuung.

* 11. Werden Ihre Intensiv-/ IMC-Stationen regelmäßig durch Apotheker/innen pharmazeutisch betreut? Zum Beispiel durch die Teilnahme an Visiten, konsiliarische Tätigkeiten, Medikationsanamnese oder regelmäßige Interaktionschecks.

- [ ] JA
- [ ] NEIN

Wenn JA, wie viele Betten durch wie viele Apotheker/innen (VK)?

Apotheker/innen auf Intensivstation

Apotheker/innen auf der Intensivstation

Diese Seite bezieht sich auf die Tätigkeit der Apotheker/innen auf der Intensivstation bzw. umfasst Fragen zur pharmazeutischen Betreuung.

12. Welche Fachrichtungen werden regelmäßig durch Apotheker/innen betreut? (Mehrfachantworten möglich)

- ☐ internistische Intensivmedizin (z.B. Gastroenterologie, Pneumologie)
- ☐ nicht-operative Intensivmedizin (z.B. Neurologie, Kardiologie)
- ☐ anästhesiologische und chirurgische Intensivmedizin
- ☐ interdisziplinäre Intensivmedizin (nicht-operativ)
- ☐ interdisziplinäre Intensivmedizin (operativ)
- ☐ Überwachungsstation(IMC)/Stroke Units
- ☐ pädiatrische Intensivmedizin (inkl. Neonatologie)
- ☐ Sonstiges (bitte angeben)

13. Welche Tätigkeiten führen Apotheker/innen im Rahmen der pharmazeutischen Betreuung durch? (Mehrfachantworten möglich)

- ☐ Bereitstellung von Arzneimittelinformation für medizinisches und pflegerisches Personal
- ☐ Medication Reconcilliation (Überprüfung der Medikation auf Vollständigkeit)
- ☐ Regelmäßige Evaluation der Medikation hinsichtlich Indikation, Dosis, Interaktion, Anamnese etc.
- ☐ Überwachung der Medikation hinsichtlich Nebenwirkung, Effektivität und Kosten
- ☐ Interventionen, die zur Therapieumstellung, Eskalation oder Deeskalation führen - z.b. durch interaktive Teilnahme an Visiten
- ☐ Therapeutisches Drugmonitoring UND Beratung (z.B. Antiepileptika und Antiinfektiva)
- ☐ Sonstiges (bitte angeben)
- ☐ Therapeutisches Drugmonitoring OHNE Beratung (z.B. Antiepileptika und Antiinfektiva)
- ☐ Empfehlungen zur Ernährungstherapie bzw. Bereitstellung individueller Regime/Konzepte
- ☐ Teilnahme ODER Durchführung von Schulungen/Fortbildung im Bereich Intensivmedizin/ IMC
- ☐ Involvierung (aktiv) in Forschungsaktivitäten in der Intensivmedizin
- ☐ Telefonische Konsultation der Apotheker/innen ist rund um die Uhr verfügbar
- ☐ Sind Apotheker/innen in das CIRS/Risikomanagement der Intensivmedizin / IMC eingebunden?

14. Wie häufig nehmen Apotheker/innen an Visiten in der Intensivmedizin/ IMC-Station teil?

- ☐ täglich
- ☐ 2 -3 mal pro Woche
- ☐ 1 mal pro Woche
- ☐ gar nicht
- ☐ auf Anfrage

15. Wie viel Zeit verbringen die Apotheker/innen im Rahmen der pharmazeutischen Betreuung in der Intensivmedizin/auf IMC-Station. Bitte schätzen Sie die Dauer in Stunden pro Tag ab.

- ☐ weniger als 0,5 h
- ☐ 0,5 - 2 h
- ☐ 2 -5 h
- ☐ mehr als 3 h

Apotheker/innen auf Intensivstation

Zusammenarbeit zwischen Apotheker/innen und der Intensivstation

* 16. Wer sind die häufigsten Ansprechpartner für Apotheker/innen auf Intensiv oder IMC-Station? (Mehrfachantworten möglich)

- [ ] Chefärztin/Chefarzt
- [ ] Fachärztin/Facharzt
- [ ] Pflegemitarbeiter/innen
- [ ] Oberärztin/Oberarzt
- [ ] Assistenzärztin/Assistenzarzt

* 17. Wie erfolgt in der Regel die Kommunikation der Apotheker/innen im Rahmen der pharmazeutischen Betreuung? (Mehrfachantworten möglich)

- [ ] mündlich (persönlich OHNE Dokumentation in der Akte, z.B. Teilnahme an Visiten)
- [ ] mündlich UND schriftlich in der Akte der Patienten
- [ ] schriftlich z.B. durch Konsile
- [ ] telefonisch

* 18. Sind die Apotheker/innen regelmäßig an der Erstellung/Erarbeitung von Klinik/Stationsstandards/Behandlungsstandards bzw. SOPs oder Verfahrensanweisungen beteiligt?

- [ ] JA
- [ ] NEIN
- [ ] Weiß ich nicht.

* 19. Sind die Apotheker/innen regelmäßig an der Erstellung von Budget- und Kosten-Nutzen Analysen beteiligt?

- [ ] JA
- [ ] NEIN
- [ ] Weiß ich nicht.

20. Die für Sie zuständigen Apotheker/innen betreuen ausschließlich die Intensivstationen/IMC?

- [ ] JA
- [ ] NEIN
- [ ] Weiß ich nicht.

21. Über wie viele Jahre Berufserfahrung verfügen die für Sie zuständigen Apotheker/innen?

- [ ] < 5 Jahre
- [ ] 5 - 10 Jahre
- [ ] > 10 Jahre
- [ ] Weiß ich nicht.

Apotheker/innen auf Intensivstation

Anforderungen an die pharmazeutische Betreuung.

* 22. Welche Tätigkeiten wünschen Sie sich und wie gewichten Sie die Arbeit der Apotheker/innen auf der Station? Bitte antworten Sie auch, falls Sie noch nicht durch Apotheker/innen regelmäßig aktiv betreut werden.

	essenziell/unverzichtbar	wünschenswert	optional
Bereitstellung von Arzneimittelinformation	◯	◯	◯
Teilnahme an Visiten	◯	◯	◯
Überprüfung der Medikation auf Vollständigkeit (MedRec)	◯	◯	◯
Regelmäßige Evaluation der Verordnung (Indikation, Dosis,Interaktion)	◯	◯	◯
Überwachung der Medikation (Nebenwirkung, Effektivität, Kosten)	◯	◯	◯
Interventionen, die zur Therapieumstellung ggfs Deeskalation führen	◯	◯	◯
Therapeutisches Drugmonitoring UND Beratung	◯	◯	◯
Therapeutisches Drugmonitoring OHNE Beratung	◯	◯	◯
Empfehlungen zur Ernährungstherapie bzw Bereitstellung individueller Regime/Konzepte	◯	◯	◯
Teilnahme UND/ODER Durchführung von Schulungen/Fortbildungen in der Intensivmedizin	◯	◯	◯
Aktive Teilnahme an Forschungsaktivitäten (z.B. Studien)	◯	◯	◯
Telefonische Erreichbarkeit des Apothekers (24h)	◯	◯	◯
Einbindung in das CIRS/Risikomanagement	◯	◯	◯

23. Wie häufig sollten Apotheker/innen Ihrer Meinung nach an der Visite teilnehmen/auf der Station präsent sein?

☐ täglich
☐ 2-3 mal pro Woche
☐ 1 mal pro Woche
☐ gar nicht
☐ auf Anfrage

24. Falls noch keine Apotheker/innen an Visiten teilnehmen bzw. auf Ihrer Station präsent sind, soll eine solche Visitenteilnahme/Präsenz eingeführt werden?

- [ ] JA, eine Einführung ist geplant.
- [ ] NEIN, eine Einführung ist nicht geplant.
- [ ] Weiß ich nicht.

* 25. Gibt es in Ihrem Haus bereits standardisierte Vorschriften/Empfehlungen für:

	JA	NEIN	Weiß ich nicht.
die Zubereitung von Perfusoren	○	○	○
die Dosierungen bei Niereninsuffizienz (inkl.Dialyse)	○	○	○
die Dosierungen bei Leberinsuffizienz	○	○	○
die initiale Antiinfektikatherapie (inkl. Dosierungen)	○	○	○
die Vermeidung von Inkompatibilitäten bei parenteraler Applikation	○	○	○

Sonstiges (bitte angeben)

* 26. Erhalten Sie bereits aus der Apotheke vorbefüllte Perfusorspritzen? Wenn ja, bitte listen Sie die Wirkstoffe.

- [ ] JA
- [ ] NEIN
- [ ] Bitte listen Sie die Wirkstoffe:

* 27. Findet in Ihrem Haus bzw. in der Intensivmedizin eine patientenindividuelle UNIT DOSE Versorgung statt?

- [ ] JA, im gesamten Krankenhaus (inkl. Intensivmedizin)
- [ ] JA, im gesamten Krankenhaus (außer Intensivmedizin)
- [ ] JA, auf einzelnen Stationen im Krankenhaus
- [ ] NEIN, es findet KEINE Unit Dose Versorgung statt

<u>Vielen Dank,</u>

<u>dass Sie sich die Zeit genommen haben!</u>
