# Supplementary material for: Drug therapy safety supported by interprofessional collaboration between ICU physicians and clinical pharmacists in critical care units in Germany: Results of a survey
Source: Med Klin Intensivmed Notfmed. 2022 Mar 8;118(2):141–8. [Article in German] doi: 10.1007/s00063-022-00898-5 (PMC9992023; doi:10.1007/s00063-022-00898-5)
Supplement: Supplementary file 2 [file 63_2022_898_MOESM2_ESM.pdf]

Zusatzmaterial Literatur -Recherche PUBMED:

PMID,Title,Authors,Citation,First Author,Journal/Book,Publication Year<br>(critical care[MeSH Terms]) AND (pharmacist[MeSH Terms])07.09.2021
34279579,"Perspectives from the frontline: A pharmacy department's response to the COVID-19 pandemic","Collins CD, West N, Sudekum DM, Hecht JP.","Am J Health Syst Pharm. 2020 Aug 20
34279575,"COVID-19 pandemic preparedness: A practical guide from clinical pharmacists' perspective","Ahuja T, Merchan C, Arnouk S, Cirrone F, Dabestani A, Papadopoulos J.","Am J Health Syst Pharm. 2020 Sep 4
33516718,"[Contribution of a hospital pharmacy team to critical care of patients infected with SARS-CoV-2]","Besson C, Chareyre S, Kirouani N, Jean-Jean S, Bretagnolle C, Henry A, Leboucher G, Charpiat B.","Ann Pharm Fr. 2021 Jul
33438981,"Action Plan for Successful Implementation of Optimal ICU Pharmacist Activities: Next Steps for the Critical Care Pharmacist Position Paper","Murray B, Buckley MS, Newsome AS.","Crit Care Med. 2021 Feb 1
33274747,"Rising to the challenge: Pharmacy residents on the front lines during COVID-19 pandemic","Uchida E, Long-Fazio B, Marshall J, Fortier C.","Am J Health Syst Pharm. 2020 Dec 4
33166192,"Impact of a Pharmacist-Led Intensive Care Unit Sleep Improvement Protocol on Sleep Duration and Quality","Andrews JL, Louzon PR, Torres X, Pyles E, Ali MH, Du Y, Devlin JW.","Ann Pharmacother. 2021 Jul
33124653,"Justification of the value of critical care pharmacists: Still a work in progress?","Erstad BL.","Am J Health Syst Pharm. 2020 Oct 30
33038310,"The role of the critical care pharmacist during the COVID-19 pandemic","Lemtiri J, Matusik E, Cousein E, Lambiotte F, Elbeki N.","Ann Pharm Fr. 2020 Nov
32844673,"Caring for the COVID Patient: A Clinical Pharmacist's Perspective","Erstad BL.","Ann Pharmacother. 2021 Mar
32789461,"The pharmacist's role in implementation of the ABCDEF bundle into clinical practice","Schmidt LE, Patel S, Stollings JL.","Am J Health Syst Pharm. 2020 Oct 14
32620973,"ICU pharmacist describes challenges in COVID-19 care","Traynor K.","Am J Health Syst Pharm. 2020 Jul 23
32533669,"Hospital Pharmacy in the multidisciplinary team of COVID inpatient units","Gil-Navarro MV, Luque-Márquez R.","Farm Hosp. 2020 Jun 12
32533667,"Hospital Pharmacist experience in the Intensive Care Unit: Plan COVID","García-Gil M, Velayos-Amo C.","Farm Hosp. 2020 Jun 12
32487967,"Interventions to ensure medication safety in acute care: an umbrella review","Khalil H, Kynoch K, Hines S.","Int J Evid Based Healthc. 2020 Jun
32468327,"Neurocritical Care Resource Utilization in Pandemics: A Statement by the Neurocritical Care Society","Moheet AM, Shapshak AH, Brissie MA, Abulhasan YB, Brophy GM, Frontera J, Hall WR, John S, Kalanuria AA, Kumar A, Lele AV, Mainali S, May CC, Mayer SA, McCredie V, Silva GS, Singh JM, Steinberg A, Sung G, Tesoro EP, Yakhkind A.","Neurocrit Care. 2020 Aug
32369877,"Intervention study for the reduction of medication errors in elderly trauma patients","Parro Martín MLÁ, Muñoz García M, Delgado Silveira E, Martín-Aragón Álvarez S, Bermejo Vicedo T.","J Eval Clin Pract. 2021 Feb
32280966,"Evidence of burnout in critical care pharmacists","Ball AM, Schultheis J, Lee HJ, Bush PW.","Am J Health Syst Pharm. 2020 May 7
32145656,"Improving medication safety in the Intensive Care by identifying relevant drug-drug interactions - Results of a multicenter Delphi study","Bakker T, Klopotowska JE, de Keizer NF, van Marum R, van der Sijs H, de Lange DW, de Jonge E, Abu-Hanna A, Dongelmans DA
32122440,"An investigation into the number and nature of the urgent care consultations managed and referred by community pharmacists in South-East England","Dodds L, Katusiime B, Shamim A, Fleming G, Thomas T.","Prim Health Care Res Dev. 2020 Mar 3
32070269,"Interventions of a clinical pharmacist in a medical intensive care unit - A retrospective analysis","Cvikl M, Sinkovič A.","Bosn J Basic Med Sci. 2020 Feb 10
31738262,"The authors reply","Lee H, Suh GY, Kim E.","Crit Care Med. 2019 Dec
31738261,"Pharmacists Are Associated With Reduced Mortality in Critically Ill Patients: Now What?","Newsome AS, Jones TW, Smith SE.","Crit Care Med. 2019 Dec
31728749,"A survey of antibiotic administration practices involving patients with sepsis in UK critical care units","Barton GJ, Morecroft CW, Henney NC.","Int J Clin Pharm. 2020 Feb
31705641,"Nationwide current situation of hospital pharmacists in intensive care units","Valera-Rubio M, Domingo-Chiva E, Aquerreta-González I, Periáñez-Párraga L, Ruiz-Ramos J, Soy-Muner D.","Farm Hosp. 2019 Nov 1

31694386,"Pathophysiology of Volume Administration in Septic Shock and the Role of the Clinical Pharmacist","Bissell BD, Mefford B.","Ann Pharmacother. 2020 Apr
31595449,"Integrating a pharmacist into an anaesthesiology and critical care department: Is this worthwhile?","Chapuis C, Albaladejo P, Billon L, Catoire C, Chanoine S, Allenet B, Bouzat P, Bedouch P, Payen JF.","Int J Clin Pharm. 2019 Dec
31544470,"Clinical Pharmacist-Led Impact on Inappropriate Albumin Use and Costs in the Critically Ill","Buckley MS, Knutson KD, Agarwal SK, Lansburg JM, Wicks LM, Saggar RC, Richards EC, Kopp BJ, Erstad BL.","Ann Pharmacother. 2020 Feb
31532504,"Select topics in the management of critically ill children","Hughes K, Buenger L.","Am J Health Syst Pharm. 2019 Sep 16
31415315,"The Bedside Critical Care Pharmacist: A Mandatory ICU Team Member Essential for Patient Care","MacLaren R, Devlin JW.","Crit Care Med. 2019 Sep
31332648,"A new approach on assessing clinical pharmacists' impact on prescribing errors in a surgical intensive care unit","Kessemeier N, Meyn D, Hoeckel M, Reitze J, Culmsee C, Tryba M.","Int J Clin Pharm. 2019 Oct
31301641,"Moral distress in intensive care unit personnel is not consistently associated with adverse medication events and other adverse events","Dodek P, Norena M, Ayas N, Dhingra V, Brown G, Wong H.","J Crit Care. 2019 Oct
31192553,"Nutrition support for critically ill patients in China: role of the pharmacist","Zhou X, Qiu F, Wan D, Sun S, Yao G, Liu Y, Li J.","Asia Pac J Clin Nutr. 2019
31175567,"Worldwide Organization of Neurocritical Care: Results from the PRINCE Study Part 1","Suarez JI, Martin RH, Bauza C, Georgiadis A, Venkatasubba Rao CP, Calvillo E, Hemphill JC 3rd, Sung G, Oddo M, Taccone FS, LeRoux PD
31135496,"Impact on Patient Outcomes of Pharmacist Participation in Multidisciplinary Critical Care Teams: A Systematic Review and Meta-Analysis","Lee H, Ryu K, Sohn Y, Kim J, Suh GY, Kim E.","Crit Care Med. 2019 Sep
30637792,"Pharmacotherapy Rounds: When Old Drugs Require New Guidelines","DeVane CL.","Pharmacotherapy. 2019 Jan
30623457,"The Optimal Use of the Polymyxins Before Their Time Is Up","Alosaimy S, Jorgensen SCJ, Rybak MJ.","Pharmacotherapy. 2019 Jan
30355597,"Perceptions of critical care pharmacists participating in a formal mentor-mentee program","Hammond DA, Holt TR, Harris SA, Caylor MM, Kopp BJ, Hamblin S.","Am J Health Syst Pharm. 2018 Nov 1
30253681,"Medication safety in a German telemedicine centre: Implementation of a telepharmaceutical expert consultation in addition to existing tele-intensive care unit services","Amkreutz J, Lenssen R, Marx G, Deisz R, Eisert A.","J Telemed Telecare. 2020 Jan-Feb
30187762,"Comment: Critical Care Pharmacists and Medication Management in an ICU Recovery Center","Wanat MA, Fitousis K.","Ann Pharmacother. 2019 Jan
30175590,"Reply: Critical Care Pharmacists and Medication Management in an ICU Recovery Center","Stollings JL, Bloom SL, Sevin CM.","Ann Pharmacother. 2019 Jan
30173635,"Hospital Pharmacy and Critical Care Medicine: a necessary alliance","Martin-Delgado MC, Calleja-Hérnandez MÁ.","Farm Hosp. 2018 Sep 1
29523535,"Association between postgraduate year 2 residency program characteristics and primary project publication","Swanson JM, Shafeeq H, Hammond DA, Li C, Devlin JW.","Am J Health Syst Pharm. 2018 Mar 15
29457491,"Critical Care Pharmacists and Medication Management in an ICU Recovery Center","Stollings JL, Bloom SL, Wang L, Ely EW, Jackson JC, Sevin CM.","Ann Pharmacother. 2018 Aug
29337793,"Expanding the Reach of Critical Care Pharmacists Globally","Devlin JW, McKenzie C.","Crit Care Med. 2018 Feb
29314430,"Reducing medication errors in critical care patients: pharmacist key resources and relationship with medicines optimisation","Bourne RS, Shulman R, Jennings JK.","Int J Pharm Pract. 2018 Dec
29024199,"Critical care pharmacy workforce: UK deployment and characteristics in 2015","Borthwick M, Barton G, Bourne RS, McKenzie C.","Int J Pharm Pract. 2018 Aug
28982305,"Advanced Modes of Mechanical Ventilation: Introduction for the Critical Care Pharmacist","Cawley MJ.","J Pharm Pract. 2019 Apr
28874082,"Impact of Multiple Daily Clinical Pharmacist-Enforced Assessments on Time in Target Sedation Range","Lizza BD, Jagow B, Hensler D, Cooper CJ, Short EJ, Maas MB, Naidech AM, Wunderink RG.","J Pharm Pract. 2018 Oct
28453820,"Reliability of clinical impact grading by healthcare professionals of common prescribing error and optimisation cases in critical care patients","Bourne RS, Shulman R, Tomlin M, Borthwick M, Berry W, Mills GH.","Int J Qual Health Care. 2017 Apr 1

28357623,"Impact of pharmaceutical care on pain and agitation in a medical intensive care unit in Thailand","Dilok-pattanamongkol P, Tangsujaritvijit V, Suansanae T, Suthisisang C.","Int J Clin Pharm. 2017 Jun
28183302,"The effect of the TIM program (Transfer ICU Medication reconciliation) on medication transfer errors in two Dutch intensive care units: design of a prospective 8-month observational study with a before and after period","Bosma BE, Meuwese E, Tan SS, van Bommel J, Melief PH, Hunfeld NG, van den Bemt PM.","BMC Health Serv Res. 2017 Feb 10
28012426,"Perceived safety and efficacy of neuromuscular blockers for acute respiratory distress syndrome among medical intensive care unit practitioners: A multicenter survey","Torbic H, Bauer SR, Personett HA, Dzierba AL, Stollings JL, Ryder LP, Daniels CE, Caples SM, Frazee EN.","J Crit Care. 2017 Apr
27699912,"PROTECTED-UK - Clinical pharmacist interventions in the UK critical care unit: exploration of relationship between intervention, service characteristics and experience level","Rudall N, McKenzie C, Landa J, Bourne RS, Bates I, Shulman R.","Int J Pharm Pract. 2017 Aug
27543598,"Impact of a pharmacist-driven protocol to decrease proton pump inhibitor use in non-intensive care hospitalized adults","Michal J, Henry T, Street C.","Am J Health Syst Pharm. 2016 Sep 1
27440623,"Comparison of medication safety systems in critical access hospitals: Combined analysis of two studies","Cochran GL, Barrett RS, Horn SD.","Am J Health Syst Pharm. 2016 Aug 1
27374959,"[Involvement of Pharmacists in Medical Care in Emergency and Critical Care Centers]","Imai T, Yoshida Y.","Yakugaku Zasshi. 2016
27179814,"Should β-lactam antibiotics be administered by continuous infusion in critically ill patients? A survey of Australia and New Zealand intensive care unit doctors and pharmacists","Cotta MO, Dulhunty JM, Roberts JA, Myburgh J, Lipman J.","Int J Antimicrob Agents. 2016 Jun
26777752,"Impact of the introduction of a specialist critical care pharmacist on the level of pharmaceutical care provided to the critical care unit","Richter A, Bates I, Thacker M, Jani Y, O'Farrell B, Edwards C, Taylor H, Shulman R.","Int J Pharm Pract. 2016 Aug
26643860,"Use of probiotics to prevent ventilator-associated pneumonia: A survey of pharmacists' attitudes","Wheeler KE, Cook DJ, Mehta S, Calce A, Guenette M, Perreault MM, Thiboutot Z, Duffett M, Burry L.","J Crit Care. 2016 Feb
26581938,"History and future of critical care pharmacy practice","Benedict N, Hess MM.","Am J Health Syst Pharm. 2015 Dec 1
26420309,"Pharmacist independent prescribing in critical care: results of a national questionnaire to establish the 2014 UK position","Bourne RS, Whiting P, Brown LS, Borthwick M.","Int J Pharm Pract. 2016 Apr
26260916,"Effect of critical care pharmacist's intervention on medication errors: A systematic review and meta-analysis of observational studies","Wang T, Benedict N, Olsen KM, Luan R, Zhu X, Zhou N, Tang H, Yan Y, Peng Y, Shi L.","J Crit Care. 2015 Oct
26077045,"Do we need a pharmacist in the ICU?","Chant C, Dewhurst NF, Friedrich JO.","Intensive Care Med. 2015 Jul
25971871,"Pharmacist's review and outcomes: Treatment-enhancing contributions tallied, evaluated, and documented (PROTECTED-UK)","Shulman R, McKenzie CA, Landa J, Bourne RS, Jones A, Borthwick M, Tomlin M, Jani YH, West D, Bates I
25907528,"Pharmacist leadership in ICU quality improvement: coordinating spontaneous awakening and breathing trials","Stollings JL, Foss JJ, Ely EW, Ambrose AM, Rice TW, Girard TD, Wheeler AP.","Ann Pharmacother. 2015 Aug
25768966,"Impact of nursing education by a pharmacist on sedation practice in a trauma surgical intensive care unit","Reinaker TS, Frock KM.","J Trauma Nurs. 2015 Mar-Apr
25666942,"Role of an electronic antimicrobial alert system in intensive care in dosing errors and pharmacist workload","Claus BO, Colpaert K, Steurbaut K, De Turck F, Vogelaers DP, Robays H, Decruyenaere J.","Int J Clin Pharm. 2015 Apr
25470782,"Expected net benefit of clinical pharmacy in intensive care medicine: a randomized interventional comparative trial with matched before-and-after groups","Claus BO, Robays H, Decruyenaere J, Annemans L.","J Eval Clin Pract. 2014 Dec
25310233,"Making decisions about medications in critically ill children: a survey of Canadian pediatric critical care clinicians","Duffett M, Choong K, Vanniyasingam T, Thabane L, Cook DJ.","Pediatr Crit Care Med. 2015 Jan
25153537,"Improving antimicrobial dosing in critically ill patients receiving continuous venovenous hemofiltration and the effect of pharmacist dosing adjustment","Jiang SP, Xu YY, Ping-Yang, Wu WF, Zhang XG, Lu XY, Xiao YH, Liang WF, Chen J.","Eur J Intern Med. 2014 Dec
24401078,"Degree of knowledge of health care professionals about pain management and use of opioids in pediatrics","de Freitas GR, de Castro CG Jr, Castro SM, Heineck I.","Pain Med. 2014 May

24256015,"[Risk management at an Intensive Care Department: conciliation of medication]","Becerril Moreno F, Bustamante Munguira E, García Verdejo JA, Bartual Lobato E, Ros Martínez M, Merino de Cós P.","Farm Hosp. 2013 Nov-Dec
24189862,"Pharmacist contributions as members of the multidisciplinary ICU team","Preslaski CR, Lat I, MacLaren R, Poston J.","Chest. 2013 Nov
24024759,"Impact of pharmacist antimicrobial dosing adjustments in septic patients on continuous renal replace-ment therapy in an intensive care unit","Jiang SP, Zhu ZY, Ma KF, Zheng X, Lu XY.","Scand J Infect Dis. 2013 Dec
24002430,"The role of the pharmacist in the intensive care unit","Jurado LV, Steelman JD.","Crit Care Nurs Q. 2013 Oct-Dec
24002429,"The impact of drug shortages on the pharmacy, nursing, and medical staff's ability to effectively care for critically ill patients","Gulbis BE, Ruiz MC, Denktas AE.","Crit Care Nurs Q. 2013 Oct-Dec
23763333,"Proactive clinical pharmacist interventions in critical care: effect of unit speciality and other fac-tors","Bourne RS, Choo CL, Dorward BJ.","Int J Pharm Pract. 2014 Apr
23719871,"BPS approves critical care, pediatrics specialty certification","Traynor K.","Am J Health Syst Pharm. 2013 Jun 15
23468188,"Clinical and financial impact of pharmacy services in the intensive care unit: pharmacist and prescriber perceptions","MacLaren R, Brett McQueen R, Campbell J.","Pharmacotherapy. 2013 Apr
23208037,"[Education of clinical pharmacy specialists in critical care in Japan]","Maeda M.","Yakugaku Zasshi. 2012
22932309,"Canadian survey of critical care pharmacists' views and involvement in clinical research","Perreault MM, Thiboutot Z, Burry LD, Rose L, Kanji S, LeBlanc JM, Carr RR, Williamson DR.","Ann Pharmacother. 2012 Sep
22354852,"Pharmacist proactive medication recommendations using electronic documentation in a UK general criti-cal care unit","Bourne RS, Choo CL.","Int J Clin Pharm. 2012 Apr
22348303,"Interventions to reduce medication errors in adult intensive care: a systematic review","Manias E, Wil-liams A, Liew D.","Br J Clin Pharmacol. 2012 Sep
22154447,"[Second wave of the French drug harmonisation programme to prevent medication errors: overall appre-ciation of healthcare professionals]","Benhamou D, Nacry R, Journois D, Auroy Y, Durand D, Arnoux A, Olier L, Castot A.","Ann Fr Anesth Reanim. 2012 Jan
21934036,"Current perceptions and practices surrounding the recognition and treatment of delirium in the intensive care unit: a survey of 250 critical care pharmacists from eight states","Devlin JW, Bhat S, Roberts RJ, Skrobik Y.","Ann Pharmacother. 2011 Oct
21275491,"Interdisciplinary patient care in the intensive care unit: focus on the pharmacist","Erstad BL, Haas CE, O'Keeffe T, Hokula CA, Parrinello K, Theodorou AA.","Pharmacotherapy. 2011 Feb
20445206,"End-of-life issues: an occupational therapy and pharmacy perspective","Reyner K.","Am J Hosp Palliat Care. 2010 May
20164471,"Evaluation of the impact of a tele-ICU pharmacist on the management of sedation in critically ill mechani-cally ventilated patients","Forni A, Skehan N, Hartman CA, Yogaratnam D, Njoroge M, Schifferdecker C, Lilly CM.","Ann Pharmacother. 2010 Mar
20065268,"Reliability and accuracy of practitioner-calculated Acute Physiology and Chronic Health Evaluation II scores for determining the appropriateness of drotrecogin alfa (activated)","Owen PS, Tan EC, Kiser TH, Fish DN, MacLaren R.","Am J Health Syst Pharm. 2010 Jan 15
19996586,"Implementation of practice guidelines for antifungal therapy in a surgical intensive care unit and its im-pact on use and costs","Swoboda S, Lichtenstern C, Ober MC, Taylor LA, Störzinger D, Michel A, Brobeil A, Mieth M, Hofer S, Sonntag HG, Hoppe-Tichy T, Weigand MA.","Chemotherapy. 2009
19792995,"Key articles and guidelines relative to intensive care unit pharmacotherapy: 2009 update","Erstad BL, Bro-phy GM, Martin SJ, Haas CE, Devlin JW, Welage LS, Dager WE.","Pharmacotherapy. 2009 Oct
19558249,"Effects of pharmacist participation in intensive care units on clinical and economic outcomes of critically ill patients with thromboembolic or infarction-related events","MacLaren R, Bond CA.","Pharmacotherapy. 2009 Jul
19386940,"Preliminary national survey of pharmacist involvement in trauma resuscitation","Hale LS, Nyberg SM, Mohr AM, Wegner-Busch EK.","Am J Health Syst Pharm. 2009 May 1
19272541,"Perceived barriers to the use of sedation protocols and daily sedation interruption: a multidisciplinary survey","Tanios MA, de Wit M, Epstein SK, Devlin JW.","J Crit Care. 2009 Mar
19020439,"Clinical pharmacist on intensive care unit saves lives and reduces costs","Knibbe CA, Tjoeng MM.","Crit Care Med. 2008 Dec
19017823,"A primer on critical care pharmacy services","Erstad BL.","Ann Pharmacother. 2008 Dec
18936700,"Clinical and economic outcomes of involving pharmacists in the direct care of critically ill patients with infections","MacLaren R, Bond CA, Martin SJ, Fike D.","Crit Care Med. 2008 Dec

17987280,"International critical care hospital pharmacist activities","LeBlanc JM, Seoane-Vazquez EC, Arbo TC, Dasta JF.","Intensive Care Med. 2008 Mar
17725263,"Dangerous abbreviations: ""U"" can make a difference!","Koczmara C, Jelincic V, Dueck C.","Dynamics. 2005 Fall
17723113,"Practical considerations when developing guidelines for managing critical bleeding","Rebuck JA.","Pharmacotherapy. 2007 Sep
17723112,"Key concepts in the management of difficult hemorrhagic cases","MacLaren R.","Pharmacotherapy. 2007 Sep
17723109,"Update on transfusion medicine","Shander A, Goodnough LT.","Pharmacotherapy. 2007 Sep
17723108,"Causes and consequences of critical bleeding and mechanisms of blood coagulation","Zimmerman LH.","Pharmacotherapy. 2007 Sep
17536876,"Incidence and nature of medication errors in neonatal intensive care with strategies to improve safety: a review of the current literature","Chedoe I, Molendijk HA, Dittrich ST, Jansman FG, Harting JW, Brouwers JR, Taxis K.","Drug Saf. 2007
16679898,"A prospective study of the impact of a critical care pharmacist assigned as a member of the multidisciplinary burn care team","Patel NP, Brandt CP, Yowler CJ.","J Burn Care Res. 2006 May-Jun
16482393,"How to increase return on investment of the intensive care pharmacist - fear of flying","Hartmann M, Meier-Hellmann A.","Intensive Care Med. 2006 Apr
16477202,"The critical care clinical pharmacist: evolution of an essential team member","Horn E, Jacobi J.","Crit Care Med. 2006 Mar
16278319,"Prevention of venous thromboembolism in the medically ill","Brouse SD.","Am J Health Syst Pharm. 2005 Nov 15
12870489,"National quality forum endorses four new patient safety practices","Rollins G.","Rep Med Guidel Outcomes Res. 2003 May 16
12432975,"The critical care pharmacist: an essential intensive care practitioner","Papadopoulos J, Rebuck JA, Lober C, Pass SE, Seidl EC, Shah RA, Sherman DS.","Pharmacotherapy. 2002 Nov
9184706,"The effect of ICU sedation guidelines and pharmacist interventions on clinical outcomes and drug cost","Devlin JW, Holbrook AM, Fuller HD.","Ann Pharmacother. 1997 Jun
8205822,"The critical care pharmacist: what you get is more than what you see","Dasta JF, Jacob J.","Crit Care Med. 1994 Jun
7919577,"New (renamed) critical care journal","Vaughan LM.","Ann Pharmacother. 1994 Jun
1611166,"Critical care pharmacy update","Dasta JF.","Ann Pharmacother. 1992 Jun
1529986,"Multidisciplinary drug-use evaluation for stress ulcer prophylaxis","Hatton J.","Am J Hosp Pharm. 1992 Jun
1815447,"Pharmacist involvement in Society of Critical Care Medicine","Dasta JF, Jacobi J.","DICP. 1991 Dec
1905438,"Pharmacist interventions improve fluid balance in fluid-restricted patients requiring parenteral nutrition","Broyles JE, Brown RO, Vehe KL, Nolly RJ, Luther RW.","DICP. 1991 Feb
2125394,"Pharmacoeconomics: therapeutic and economic considerations in treating the critically ill patient","Crane VS.","DICP. 1990 Nov
3847770,"Clinical pharmacist: emerging member of the NICU team","Bryant BG.","Neonatal Netw. 1985 Jun
14014633,"What the pharmacist should do to help intensive patient care","BOWLES G Jr.","Mod Hosp. 1963 Jan
PMID,Title,Authors,Citation,First Author,Journal/Book,Publication Year<br>(critical care[MeSH Terms]) AND (pharmacy[MeSH Terms]) 07.09.2021
32233830,"Pharmacy Personnel's Involvement in Transitions of Care of Intensive Care Unit Patients: A Systematic Review","Rice M, Lear A, Kane-Gill S, Seybert AL, Smithburger PL.","J Pharm Pract. 2021 Feb
29523535,"Association between postgraduate year 2 residency program characteristics and primary project publication","Swanson JM, Shafeeq H, Hammond DA, Li C, Devlin JW.","Am J Health Syst Pharm. 2018 Mar 15
29024199,"Critical care pharmacy workforce: UK deployment and characteristics in 2015","Borthwick M, Barton G, Bourne RS, McKenzie C.","Int J Pharm Pract. 2018 Aug
27498360,"Granting Order-Writing Privileges to Registered Dietitian Nutritionists Can Decrease Costs in Acute Care Hospitals","Phillips W, Doley J.","J Acad Nutr Diet. 2017 Jun
22932309,"Canadian survey of critical care pharmacists' views and involvement in clinical research","Perreault MM, Thiboutot Z, Burry LD, Rose L, Kanji S, LeBlanc JM, Carr RR, Williamson DR.","Ann Pharmacother. 2012 Sep
22742644,"Statin therapy in critical illness: an international survey of intensive care physicians' opinions, attitudes and practice","Shankar-Hari M, Kruger PS, Di Gangi S, Scales DC, Perkins GD, McAuley DF, Terblanche M.","BMC Clin Pharmacol. 2012 Jun 28

20633097,"Creating a shared formulary in 7 critical access hospitals","Wakefield DS, Ward MM, Loes JL, O'Brien J, Abbas N.","J Rural Health. 2010 Summer
7600823,"Critical care pharmacists: the bridge tenders","Whipple JK, Quebbeman EJ, Wallace JR, Ausman R.","Crit Care Med. 1995 Jul
7094842,"The critically ill or injured patient: priorities in management","Majerus TC.","Drug Intell Clin Pharm. 1982 Jun
